# Anticoagulants for atrial fibrillation: from warfarin and DOACs to the promise of factor XI inhibitors

**DOI:** 10.3389/fcvm.2024.1352734

**Published:** 2024-02-05

**Authors:** Vineet Kumar, Leonard Ilkhanoff

**Affiliations:** Electrophysiology Section, INOVA Fairfax Hospital, INOVA Schar Heart and Vascular Institute, Falls Church, VA, United States

**Keywords:** anticoagulant, atrial fibrillation, direct oral anticoagulants, factor XI inhibitors, arrhythmia

## Abstract

Anticoagulation is the mainstay of stroke prevention in appropriate patients with atrial fibrillation. Due to advances in pharmacotherapy the anticoagulants used for this purpose have evolved significantly over the past decades with the aim of optimizing effectiveness while minimizing bleeding risks. Though significant improvements have been made toward this goal, bleeding risk remains the major concern with these therapies. An investigational class of agents which inhibit Factor XI have shown promise in pre-clinical and early clinical trials to significantly minimize bleeding while maintaining efficacy against stroke and systemic embolism. This mini-review will discuss anticoagulants currently used for stroke prevention in patients with atrial fibrillation including warfarin and direct oral anticoagulants. We will also review the mechanism of action and data from early clinical trials for Factor XI inhibitors and discuss their potential advantages and shortcomings.

## Introduction

Atrial fibrillation (AF) is the most prevalent arrhythmia in the world impacting over 46 million individuals. AF is a frequent and major cause of stroke, affecting approximately 800,000 individuals in the United States annually, of which three-quarters are new strokes, leading to significant healthcare resource expenditure (1). Most strokes associated with AF are cardioembolic, precipitated by the formation of thrombus within the left atrial appendage which then embolizes and impedes blood flow supplying brain tissue leading to neurologic impairment. Cardioembolic strokes related to AF have a significantly high rate of recurrence without treatment (2).

Anticoagulation is the mainstay of stroke prevention in patients with AF. Current American Heart Association and Heart Rhythm Society (AHA/HRS) guidelines recommend oral anticoagulation to reduce the risk of stroke in patients with AF with an elevated CHA_2_DS_2_VASC [congestive heart failure; hypertension, age ≥75 years; diabetes mellitus; vascular disease (prior MI, PAD, or aortic plaque), age 65–74 years; female sex] score of ≥3 for women and ≥2 for men (2). Despite a decades long history supporting anticoagulant use to reduce stroke risk, no anticoagulant to date has proven to provide absolute protection against stroke without increasing the risk of bleeding.

In this review, we highlight the history of anticoagulant use in AF management and underscore the benefits and shortcomings of existing anticoagulants for the treatment of AF. We will provide an overview of warfarin and the direct oral anticoagulants (DOACs), specifically the direct thrombin inhibitors (dabigatran), and the Factor Xa inhibitors (rivaroxaban, apixaban, and edoxaban). We will focus this review on the emerging class of Factor XI inhibitors. With growing enthusiasm surrounding these novel molecules, we will describe their potential advantages and limitations in the treatment of stroke prevention in AF and review clinical trials currently underway evaluating their efficacy and safety in AF stroke prevention.

## First generation oral anticoagulants for stroke prevention in atrial fibrillation: Warfarin and vitamin K antagonists

Historically, vitamin K antagonists (VKAs) such as warfarin were the only available oral anticoagulants used to prevent stroke in AF. Despite their widespread use, VKAs present significant pharmacologic and practical disadvantages to patients, as dosing is highly variable from patient to patient due to the unique pharmocodynamic and pharmacokinetic properties. This variability reflects drug–drug and drug–food interactions, liver metabolism, as well as genetic polymorphisms that complicate the balance between the anticoagulant effects (and therefore, stroke risk reduction) and bleeding risk and complications. Perhaps as a result of this variability, VKAs have been associated with a higher risk of both nuisance and high risk bleeding events compared to DOACS, including a higher risk of intracranial bleeding than DOACs (3, 4).

There are, however, unique advantages to warfarin in patients with AF. First, in patients with rheumatic mitral valve disease (especially in moderate to severe mitral stenosis), warfarin is the anticoagulant of choice, conferring greater protection against stroke than DOACs. Second, in patients with mechanical heart valves, warfarin is also the preferred anticoagulant over novel agents. Randomized trials have demonstrated a higher risk of thrombosis using DOACS as compared to warfarin in this setting and current guideline recommendations strongly support the use of warfarin (2).

## Second generation oral anticoagulants for stroke prevention in atrial fibrillation: prothrombin inhibitors and factor X inhibitors

**Figure 1:**
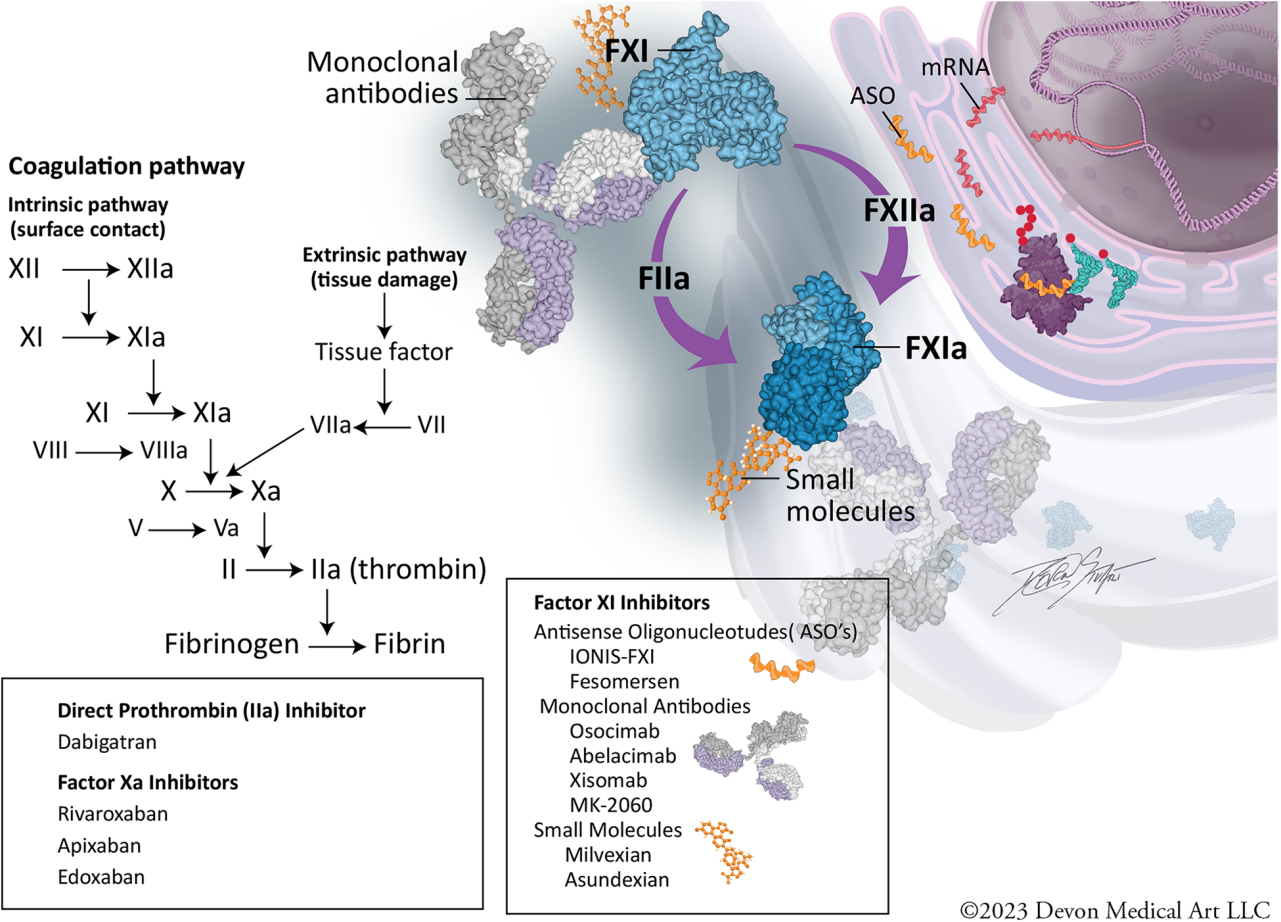
Coagulation cascade and mechanisms of inhibition.

Designed to address and potentially overcome many of the pitfalls of warfarin, DOACs leverage novel mechanisms of action (Figure 1) to inhibit and target specific factors in the coagulation cascade. Increased specificity for a single component of the coagulation cascade affords DOAC class anticoagulants significant advantages including a reduced need for frequent laboratory monitoring of anticoagulation effect.

Across four large randomized clinical trials, DOACs have demonstrated similar efficacy and safety compared to warfarin in the prevention of stroke and systemic embolism in patients with non-valvular AF (5–8). Meta-analyses of these trials also have demonstrated a relative risk reduction in all strokes and systemic embolism (19% reduction), intracranial hemorrhage (52% reduction) and all-cause death (10% reduction). However, DOACs were associated with a higher risk of gastrointestinal bleeding (25% increase) compared to warfarin (9, 10). Moreover, “real-world” data comparing DOACs to warfarin—with the inherent limitations of observational studies—demonstrate findings consistent with clinical trial data across similar outcomes (9, 10).

Overall, compared to warfarin, DOACs have a more rapid onset and offset of action, fixed dosing regimens, fewer interactions with food and drugs, and no requirements for laboratory monitoring. In addition, DOACs appear to confer greater protection against the risk of intracranial hemorrhage compared to warfarin. Taken together, current guidelines recommend DOACs over warfarin as first-line therapies for stroke prevention in patients with AF and elevated risk of stroke (2). Nevertheless, limitations of DOACs still include a modest gastrointestinal bleeding profile and a variable dependence on renal clearance, which differs by drug used.

## Third generation oral anticoagulants for stroke prevention in atrial fibrillation: factor XI inhibitors

Despite the advantages of DOACs compared to warfarin, the risk of major and minor bleeding remains a concern. A new class of pharmacotherapy, Factor XI inhibitors, aims to reduce thrombosis without significantly affecting hemostasis and may prove to be a promising strategy for stroke prevention with minimal bleeding risk in AF patients.

The coagulation cascade consists of a complex series of biochemical events occurring on cellular surfaces across three overlapping stages: initiation, amplification, and propagation. The cascade includes the intrinsic and extrinsic pathways, which converge at the common pathway to form a stable clot (Figure 1). Factor XI is part of the intrinsic pathway and circulates in an inactive form in the bloodstream. When vascular damage occurs, Factor XI is activated to Factor XIa by thrombin (initiation). Once activated, Factor XIa plays an important role in amplifying the coagulation response by activating Factor IX, which in turn, activates Factor X (amplification phase). This leads to a burst in thrombin generation, ultimately converting fibrinogen to fibrin and clot formation (propagation) (11–14).

## Rationale for drug development of factor XI inhibitors

Factor XI inhibitors disrupt the amplification of the coagulation cascade specifically by reducing thrombin generation and fibrin formation. The inhibition occurs upstream from the formation of thrombin and fibrin, making Factor XI inhibitors a novel approach to anticoagulation (12).

The rationale for the development of Factor XI inhibitors stems from multiple pre-clinical as well as clinical observations. Congenital Factor XI deficiency, also called Hemophilia C, has a variable phenotype but many patients with the disorder are asymptomatic and have little to no increased bleeding risk. In addition, patients with Factor XI deficiency have minimal bleeding often only associated with trauma. In addition to a nominal bleeding risk, patients with congenital Factor XI deficiency have lower rates of r of venous thromboembolism and cardiovascular events compared to patients with normal Factor XI activity (12, 14, 15). In preclinical studies, animal models deficient in Factor XI have reduced thrombosis with similar bleeding times (11, 12). Drugs that target Factor XI aim to deliver similar therapeutic profile, a reduction in risk forthrombotic and embolic events with little to no increase in bleeding If this class of oral anticoagulants achieves this goal, they will offer improved outcomes for currently untreated or undertreated individuals with AF who have a pre-existing high or seemingly prohibitive risk of bleeding.

## Mechanism of action of factor XI inhibitors

Factor XI inhibitors can be subdivided based on their site of action in the Factor XI synthesis and activation pathway into three broad categories: (1) Antisense oligonucleotides (ASO's) (2) Antibodies to Factor XI and XIa (3) Small molecules that bind to Factor XI and XIa.

ASO's are synthetic single stranded nucleotides which can bind and inhibit mRNA transcription thereby decreasing the level of the expressed protein (16). Their onset and offset of action is slow and are usually delivered through subcutaneous injection on a weekly or monthly basis. Although currently there are no ASO's approved for treatment of cardiovascular disease, they have been approved by FDA for treatment of muscular dystrophy (17). Ionis FXI and Fesomersen are two ASO's that inhibit Factor XI synthesis and are currently under investigation (12) for prevention of venous thromboembolism in patients undergoing orthopedic surgery. Utility of ASO's in prevention of cardioembolic stroke in patients with AF at high risk of stroke may be limited due to their relatively slow onset of action(weeks) thus requiring bridging or exposing the patient to small but not insignificant risk of stroke.

*Monoclonal antibodies* act by binding to their target proteins with high affinity and modifying/inhibiting their action. The potential utility of antibodies to Factor XI and XIa for prevention of thromboembolism is being evaluated in several clinical trials. These drugs are administered intravenously or subcutaneously on a monthly basis but have a relatively rapid onset of action compared to ASO's thereby making them more attractive for use in patients with AF. Their metabolism is independent of renal or hepatic function and thus may offer significant advantages over the currently available DOACs in patients with end stage renal disease (ESRD). They, however, can be immunogenic and therefore result in local injection site reaction, hypersensitivity and tolerance with repeated administration. This remains a concern with this class of drugs in general (18). Abelacimab (19), Osocimab (20) and Xisomab (21) are some of the monoclonal antibodies currently under development for Factor XI inhibition. Abelacimab has been studied for stroke prevention in patients with AF. It acts by binding to the active domain of Factor XI and inhibits the activity of Factor XI and its activated form Factor XIa. It has a half life of around 4 weeks thus allowing for monthly administration.

*Small molecules* are synthetic compounds which because of their low molecular weight can easily diffuse across cell membranes. They have a relatively rapid onset and offset of action and can be administered orally. Their metabolism, however, may be dependent on renal and hepatic function and thus need closer monitoring in a subset of patients who have renal or hepatic dysfunction. Asundexian (22) and Milvexian (23) are two small molecules which inhibit Factor Xia. Asundexian is currently being evaluated for stroke prevention in patients with AF at high risk of stroke. Its half-life is 15–20 h necessitating daily administration.

## Clinical trials of factor XI inhibitors in patients with atrial fibrillation

Abelacimab and Asundexian have been studied in phase 1 and phase 2 clinical trials in patients with AF and have demonstrated promising preliminary results. Phase 3 trials of these drugs are currently underway. Below we will summarize the important completed and ongoing trials of these two drugs in patients with AF.

## Abelacimab trials

ANT-004 (24) was a phase 1 trial evaluating the safety and pharmacokinetics of Abelacimab in patients with AF. Patients were administered monthly subcutaneous doses of Abelacimab (120 mg and 180 mg), or placebo, for 3 months. The results demonstrated significant and sustained reduction in free Factor XI levels with no clinically relevant bleeding leading the way for this drug to be tested in phase 2 and 3 trials.

### LILAC-TIMI 76

The (LILAC-TIMI 76 NCT05712200) is a phase 3 randomized, placebo-controlled, double-blind trial to evaluate the safety and efficacy of Abelacimab relative to placebo in patients with AF who are unable to take currently available anticoagulation therapy. The primary efficacy and safety end points are the rate of ischemic stroke and clinically relevant bleeding respectively. The trial is currently enrolling with expected completion in 2025.

### AZALEA-TIMI 71

The (AZALEA-TIMI 71 NCT04755283) trial is a phase 2 randomized trial comparing the effect of two blinded doses of Abelacimab relative to rivaroxaban on the rate of major or clinically relevant non-major (CRNM) bleeding events in patients with AF who are at moderate-to-high risk of stroke (CHADSVASC2 ≥ 4). The trial was stopped prematurely in September 2023 due to significantly lower rate of bleeding with Abelacimab.

The combined rate of major and clinically relevant nonmajor bleeding was 8.1 per 100 patient-years in the rivaroxaban group and 2.7 per 100 patient-years in the 150 mg Abelacimab group (HR 0.33; 95% CI 0.19–0.55). In the 90-mg Abelacimab group, bleeding occurred at a rate of 1.9 per 100 patient-years (HR 0.23; 95% CI 0.13–0.42) compared with Rivaroxaban.

Major bleeding occurred at a rate of 3.7 events per 100 patient-years in the rivaroxaban group vs. 1.0 per 100 patient-years in the 150-mg Abelacimab group (HR 0.26; 95% CI 0.11–0.61. Similarly, the incidence of GI bleed was significantly lower with Abelacimab (0.1 per 100 patient-years at either dose vs. 2.1 per 100 patient-years with Rivaroxaban) The trial was not designed to compare efficacy in stroke prevention among the three arms and a larger phase 3 trial will be needed.

## Asundexian trials

### PACIFIC-AF

PACIFIC-AF (8) was a phase 2 dose finding study comparing two doses of Asundexian to Apixaban in patients with AF. It demonstrated Asundexian significantly reduced Factor XI levels at both 20 mg and 50 mg dose. The level of inhibition of Factor XI at peak drug concentration was 90% with the 20 mg dose and 92% at the 50 mg dose. The trial also demonstrated significantly lower incidence of clinically relevant non major bleeding (CRNM) with Asundexian compared to Apixaban (HR 0.33, 90% CI 0.09–0.97). The trial did not compare the efficacy of the drugs for prevention of thrombotic and embolic events including cardioembolic stroke. Although the confidence intervals for reduction in bleeding were wide it was the first clinical trial to demonstrate lower rates of bleeding with Factor XI inhibitors compared to standard of care.

### OCEANIC AF

(OCEANIC AF NCT05643573) trial is part of the OCEANIC program for the development of Asundexian. This is a phase 3 multicenter double blind randomized trial comparing the efficacy and safety of Asundexian to Apixaban in patients with AF. It was supposed to enroll approximately 18,000 adult patients with AF with estimated completion in 2025. However, the trial was stopped prematurely by the recommendations of the Independent Data Monitoring Committee (IDMC) due to inferior efficacy. Full data of the trial is not available at the time of this manuscript to ascertain the reason for its inferiority to Apixaban.

## Conclusion

Factor XI inhibitors offer the promise of decoupling hemostasis from thrombosis and thereby offering a superior safety profile over current anticoagulants used for stroke prevention in AF. They have been evaluated in phase 1 and phase 2 human trials which have demonstrated consistently high levels of Factor XI inhibition without increasing bleeding. It is important to note that these preliminary yet promising results need to be validated in large phase 3 trials. Results from Oceanic AF are a sobering reminder that the efficacy of these agents needs to be validated in large Phase 3 randomized trials. It is unclear at this time if the lack of efficacy of Asundexian is peculiar to this agent due to its mechanism of action or a broader class effect. Finally, large clinical trials with longer follow-up might also alert us to any potential adverse effects from long term inhibition of Factor XI and other safety concerns which may arise from this new class of drugs. Nonetheless, the promise of anticoagulation for stroke prevention in AF continues to evolve and our pharmacologic options to protect patients from stroke while reducing their bleeding risk continues to make substantial progress.
